# Thoracic Complications in Behçet's Disease: Imaging Findings

**DOI:** 10.1155/2020/4649081

**Published:** 2020-05-28

**Authors:** Kemal Ödev, Recep Tunç, Salih Varol, Harun Aydemir, Pınar Didem Yılmaz, Celalettin Korkmaz

**Affiliations:** ^1^Department of Radiology, Konya Chamber of Commerce Karatay University, Faculty of Medicine, Konya, Turkey; ^2^Department of Rheumatology, Necmettin Erbakan University, Faculty of Medicine, Konya, Turkey; ^3^Department of Physiology, Konya Chamber of Commerce Karatay University, Faculty of Medicine, Konya, Turkey; ^4^Department of Radiology, Necmettin Erbakan University, Faculty of Medicine, Konya, Turkey; ^5^Department of Chest Clinic, Necmettin Erbakan University, Faculty of Medicine, Konya, Turkey

## Abstract

Behçet's disease (BD) causes vascular inflammation and necrosis in a wide range of organs and tissues. In the thorax, it may cause vascular complications, affecting the aorta, brachiocephalic arteries, bronchial arteries, pulmonary arteries, pulmonary veins, capillaries, and mediastinal and thoracic inlet veins. In BD, chest radiograph is commonly used for the initial assessment of pulmonary symptoms and complications and for follow-up and establishment of the response to treatment. With the advancement of helical or multislice computed tomography (CT) technologies, such noninvasive imaging techniques have been employed for the diagnosis of vascular lesions, vascular complications, and pulmonary parenchymal manifestations of BD. CT scan (especially, CT angiography) has been used to determine the presence and severity of pulmonary complications without resorting to more invasive procedures, in conjunction with gadolinium-enhanced three-dimensional (3D) gradient-echo magnetic resonance (MR) imaging with the subtraction of arterial phase images. These radiologic methods have characteristics that are complementary to each other in diagnosis of the thoracic complications in BD. 3D ultrashort echo time (UTE) MR imaging (MRI) could potentially yield superior image quality for pulmonary vessels and lung parenchyma when compared with breath-hold 3D MR angiography.

## 1. Introduction

BD is a chronic, systemic inflammatory disease of unknown origin [1–4]. A clinical triad of oral ulcers and genital ulcers and uveitis is characteristic of the disease. This triple symptom complex was first described by the Turkish dermatologist Hulusi Behçet in 1937 [5]. Clinical manifestations additional to this triad were described later including involvement of the skin, joints, large vessels, lung, brain, and gastrointestinal and genitourinary tracts (Table 1) [4, 6–8]. The variability of clinical manifestations in addition to specific histological or laboratory findings may cause the difficulty in diagnosis [8]. There are no specific diagnostic laboratory tests or histopathologic findings. Thus, the diagnosis of the disease is made on the basis of the criteria proposed by the International Study Group for Behçet's disease in 1990 (Table 2) [9]. Immunopathological findings suggest that the main underlying pathology in the patients suffering from Behçet's disease is vasculitis affecting the small and large pulmonary arteries [3, 4, 8]. In comparison with the conventional radiological techniques, helical CT or multislice CT (MSCT) is the preferred imaging modality in the diagnostic procedures related to the investigation of thoracic involvements in BD [8, 10, 11]. Undoubtedly, the need for conventional radiology has diminished with the increasing utility of CT scan. However, CT scans in association with magnetic resonance imaging (MRI) and MR angiography (MRA) or MR venography may prove useful in obtaining detailed thoracic findings of BD [12–14].

In this study we review the clinical and imaging findings of BD.

## 2. Clinical Findings

BD has a very wide spectrum of clinical features and it has a chronic course with unstable exacerbations and remissions [15]. Hemoptysis is the most common clinical manifestation of pulmonary involvement [3, 8, 16–20]. This finding is a poor prognostic sign, leading to death in 30% of patients, in 80% within two years [14, 18]. Other clinical problems may include dyspnea, pleuritic chest pain, cough, malaise, fever, and weight loss [3, 16, 18, 19]. Pulmonary artery aneurysms can be fatal due to perforation to the adjacent bronchus [3, 18–20] or it may be the result of the development of in situ thrombosis related to the active vasculitis [3, 8, 19]. In BD, mediastinal involvement with mediastinal fibrosis might be initiated by chronic relapsing systemic vasculitis. Patients with mediastinal fibrosis and superior vena cava (SVC) thrombosis present with signs or symptoms related to obstruction or compression of vital mediastinal structures such as central airways, axillar, subclavian and brachiocephalic veins, and SVC [21, 22].

Cardiovascular manifestations have been reported in 7–46% of cases with BD and mortality occurs in up to 20% of those patients with marked vascular involvement [23, 24]. Sporadic cases of endocarditis, myocarditis, pericarditis, acute myocardial infarction, aortic aneurysm, ventricular thrombosis, congestive cardiomyopathy, and valvular dysfunction have been reported [23, 25].

## 3. Imaging Findings

### 3.1. Chest Radiograph

Chest radiograph is the first choice in imaging technique to demonstrate the pulmonary signs and symptoms of BD [14]. Pulmonary artery aneurysms (PAAs) appear as a hilar enlargement or as round, lobular opacities on chest radiographs. These lesions resemble hilar mass lesions. Chest radiographs are also useful for the detection of hilar enlargement, which may develop due to PAAs [8, 14]. Chest radiograph can show the clinical effectiveness of immunosuppressive treatment and findings related to response to the treatment in early period during follow-up [26]. Radiograph may be normal or may show indirect signs such as mediastinal widening [10, 14].

### 3.2. Angiography

Contrast studies of the arteries and veins are the standard method in diagnosing vascular lesions and vascular complications in BD and for evaluating their response to medical therapy. If a source of bleeding in the bronchial artery is suspected, it may be controlled via embolization techniques during arteriography [12, 14, 27]. However, venous puncture, intravenous infusion, rapid injection of a large quantity of contrast medium, or insertion of vascular catheters may initiate the formation of thrombosis or aneurysm. Moreover, an aneurysm may develop at the site of the arterial puncture [3, 10, 14]. In the literature, following angiography for the demonstration of vascular lesions has been reported as being severely hazardous (in 70% in patients) [16, 18]. Surgical repair carries a high risk because of the presence of massive hemoptysis. Thus, arterial embolization with catheter angiography is advocated as alternative life-saving method for treatment of PAAs in BD with massive, life-threating hemoptysis [28].

### 3.3. Computed Tomography (CT)

Helical CT (spiral CT) or MSCT is an important imaging technique in demonstrating the entire thoracic manifestations in BD [10, 11, 29]. Two-dimensional and 3D reformatted chest CT images have been suggested as the most appropriate imaging techniques because contrast-enhanced CT scans can provide a surgical road map of contrast-filled lumen of major or mediastinal arteries, mediastinal and thoracic inlet veins, and segmental and larger pulmonary arteries [11, 12, 14]. When compared with contrast-enhanced CT, it has been reported that helical CT phlebography is superior to contrast-enhanced CT for showing of thrombi and for determining the degree of obstruction. Although CT phlebography shows the site and the extent of obstruction for visualizing collateral pathways, it has been reported that this technique is insufficient to diagnose the cause of obstruction [30]. Major technical limitation of CT phlebography is artifact related to administration of contrast material. The other limitation is dilution of opacified venous blood by unopacified blood. These findings may cause nonhomogeneous intraluminal opacification of normal veins [31]. MSCT or MDCT, with its multiplanar and 3D imaging, depicts mediastinal widening and identifies superimposed thrombosis, severity of SVC obstruction, and the level and extent of venous obstruction [32]. The CT diagnosis of SVC obstruction includes lack of opacification of SVC, intraluminal filling defect or severe narrowing of the SVC, and visualization of collateral vascular channels [32].

In addition, CT scan examinations (especially, CT angiography) can detect and locate aneurysms and central or segmental pulmonary thromboemboli. CT scans can also identify the origin of aneurysm and its anatomic relationships with the adjacent structures and the pulmonary artery. Moreover, it is useful in determining the need for intervention and the optimum surgical approach [12, 33]. CT is significantly more accurate than angiography in the assessment of intraluminal thrombi, wall calcification, and extraluminal disease [33]. Furthermore, angiography could not demonstrate pulmonary aneurysms if they are completely thrombosed [34]. Consequently, CT angiography also provides excellent vascular images with only a small quantity of contrast medium and may be used as an alternative to venography and angiography [10, 14].

### 3.4. Magnetic Resonance Imaging (MRI)

A review of the clinical and radiology literature has shown that MRI and MRA can be used to detect the exact localization of the aneurysm of the aorta, brachiocephalic arteries, and PAAs, as well as changes in the vessel walls, mural thrombus, and thrombus within the aneurysms [12, 14]. However, a few studies suggest that MRI is less sensitive than helical CT for the detection of small aneurysms in the lungs [20, 35].

Thrombosis or the obstruction of the central veins is an important cause of morbidity or mortality in patients with benign or malignant conditions. Several investigators [36–38] have advocated that MRI and 3D gadolinium-enhanced MR venography with the subtraction of arterial phase images can be successfully used in the evaluation of thoracic veins and obstructions of the central veins. In a study by Akpolat et al. [13], it has been reported that MRI and MRA can demonstrate not only the arterial lesions but also the venous occlusions in the central and peripheral veins in BD patients. However, the advantages and limitations of MR examinations have been well described in [37]. Because no contrast agent is needed to image flowing blood, MR examination can be performed safely in patients with allergic to iodinated contrast media or in those for whom such media are contraindicated medically. Gadolinium-based contrast agents include risk of nephrotoxicity in patients with renal insufficiency and pregnant patients or in patients in whom intravenous access cannot be obtained. In these patients alternative imaging techniques such as noncontrast MR venography (phase contrast MRA) can be used to evaluate the venous lesions [39–41]. Some researchers [42–44] have advocated that free-breathing 3D radial ultrashort echo time (UTE) MRI can be used in both detection of lung parenchymal lesions (e.g., emphysema and fibrosis) and pulmonary vascular pathologies (e.g., pulmonary emboli) simultaneously. According to the literature reports, several types of thoracic complications associated with BD have been described in the literature [10, 11].

## 4. Involvement of Thoracic Vessels

### 4.1. Systemic Arterial Manifestations

**Figure 1:**
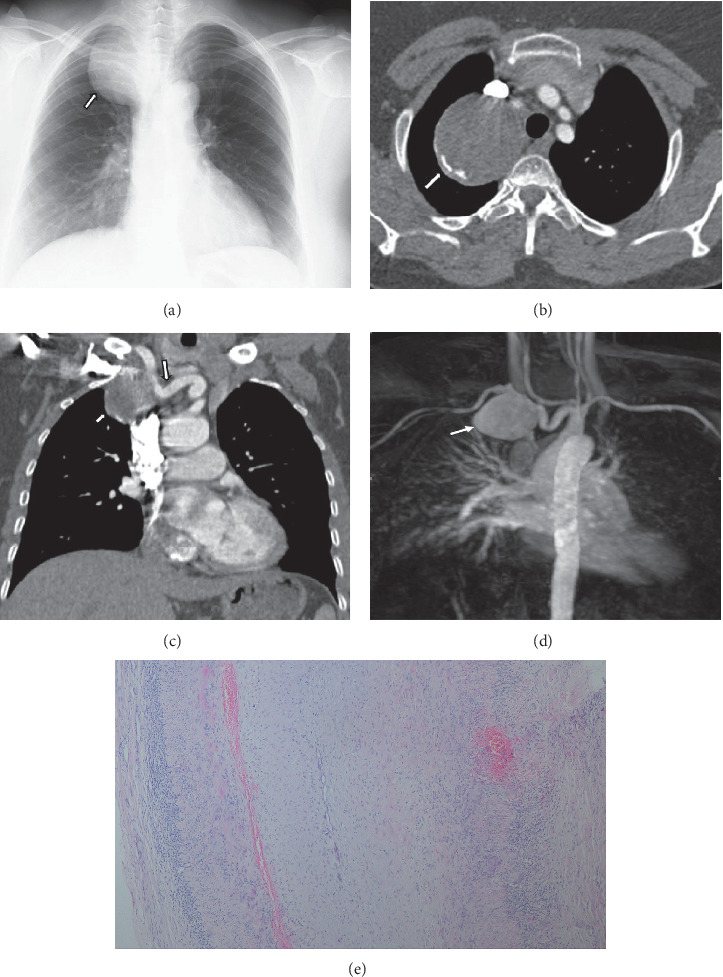
A 45-year-old woman with chronic cough, dyspnea, and hemoptysis. She had no family history of Behçet's disease or familial vascular disorder. (a) Chest radiograph showing a focal right paratracheal mass (white arrow). (b) Axial contrast-enhanced chest CT scan (mediastinal window) at the level of the thoracic inlet showing a mass with curvilinear calcification (white arrow). (c) Coronal contrast-enhanced chest CT scan (mediastinal window) showing a round mass (white arrow) in contact with the right subclavian artery (arrow head). Curvilinear calcification is visible in the wall of the mass. (d) Coronal maximum-intensity projection magnetic resonance angiography image showing a saccular aneurysm at the right subclavian artery (white arrow). The diagnosis of Behçet's disease was made after histopathological examination of the resected specimen following surgery. (e) Histopathology of resected subclavian arterial aneurysm. Hematoxylin-eosin, magnification x40. Chronic inflammatory process containing lymphocyte infiltration was detected in the adventitia that was compatible with diagnosis of Behçet's disease.

The entire arterial tree can be involved in BD. Although vascular involvement is noted in only 25% of all affected patients, it is the most common cause of mortality in BD [10, 14, 18]. BD associated with lesions in the large vessels is referred to as ‘vasculo-BD' or ‘angio-BD' [4, 10]. The most frequent site of systemic arterial involvement is the thoracic aorta, brachiocephalic arteries, subclavian artery (Figure 1) abdominal aorta, and peripheral arteries. Involvement of visceral vessels occurs rarely. The arterial lesions are aneurysm, stenosis, complete occlusion, and pseudoaneurysms [14, 45, 46]. Arterial occlusions or stenosis may be asymptomatic or associated with ischemic symptoms, depending on the site of involvement and collateral circulation. The pathogenesis of the arterial lesions is thought to be related to vasa vasorum and arterial wall vasculitis [47]. Differential diagnosis of the vascular lesions includes Takayasu's arteritis, connective tissue disease, ankylosing spondylosis, Reiter's disease, and Burger disease [46, 47].

### 4.2. Pulmonary Artery Involvement

**Figure 2:**
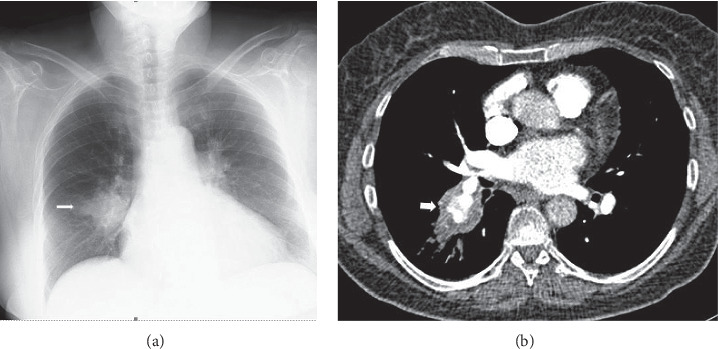
A 40-year-old woman with pulmonary artery aneurysm who had hemoptysis and chest pain. (a) Initial chest radiograph showing a round opacity at the inferior portion of the hilus on the right (arrow). (b) Axial contrast-enhanced chest CT scan (curved multiplanar reconstruction) (mediastinal window) showing a saccular aneurysm of the right interlobar artery with a circumferential mural thrombus (arrow).

**Figure 3:**
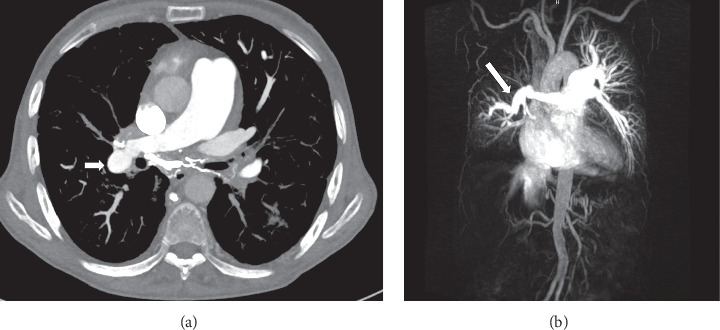
Pulmonary artery aneurysm in a 37-year-old male with hemoptysis. (a) Axial contrast-enhanced CT scan (curved multiplanar reconstruction) (mediastinal window) showing an aneurysm of the right interlobar pulmonary artery (arrow). (b) MIP-reconstructed MRA image demonstrates an aneurysm of the right interlobar pulmonary artery aneurysm (long arrow).

**Figure 4:**
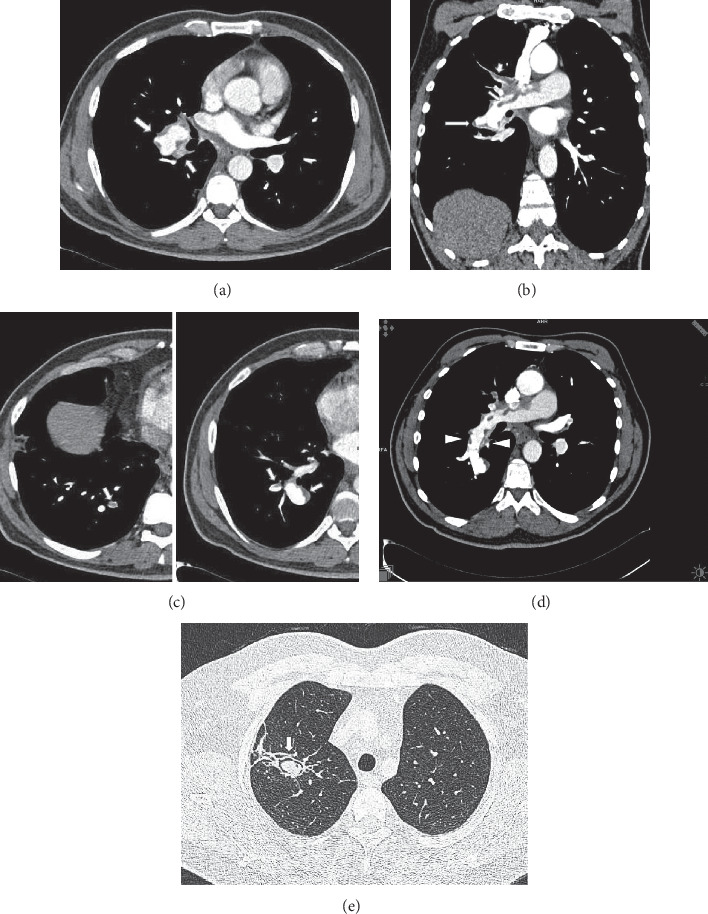
A 40-year-old man with pulmonary artery aneurysms who had recurrent hemoptysis and chest pain and dyspnea. (a) Before treatment with immunosuppressive axial contrast-enhanced chest CT scan (mediastinal window) showing an aneurysm with lobulated contour on the right interlobar pulmonary artery (arrows). (b) Follow-up coronal maximum-intensity projection CT image showing multiple intraluminal filling defects within the upper lobe segmental branches of the right pulmonary artery (short arrows) and the right pulmonary artery with lobulated contour (arrow). (c) An axial contrast-enhanced CT scan through the lower chest showing basal segmental pulmonary arteries with central filling defects consistent with thromboembolism (arrows). *Note.* In this patient, deep vein thrombophlebitis and deep vein thrombosis in the lower extremities were not observed by Doppler ultrasonography. Thus, pulmonary thromboemboli probably developed as a complication of anticoagulant therapy. (d) Three years after treatment with immunosuppressive, axial maximum-intensity projection CT (curved multiplanar reconstruction) image shows regression of aneurysm on the right interlobar pulmonary artery (arrow heads). (e) High-resolution CT scan obtained 1-year later following immunosuppressive treatment showing a soft tissue nodule with air-crescent sign (arrow) within a cavity in the right upper lung and mycetoma showing a characteristic crescent of air between the mycetoma and the cavity wall. Radiological and serological tests confirmed *Aspergillus fumigatus*.

**Figure 5:**
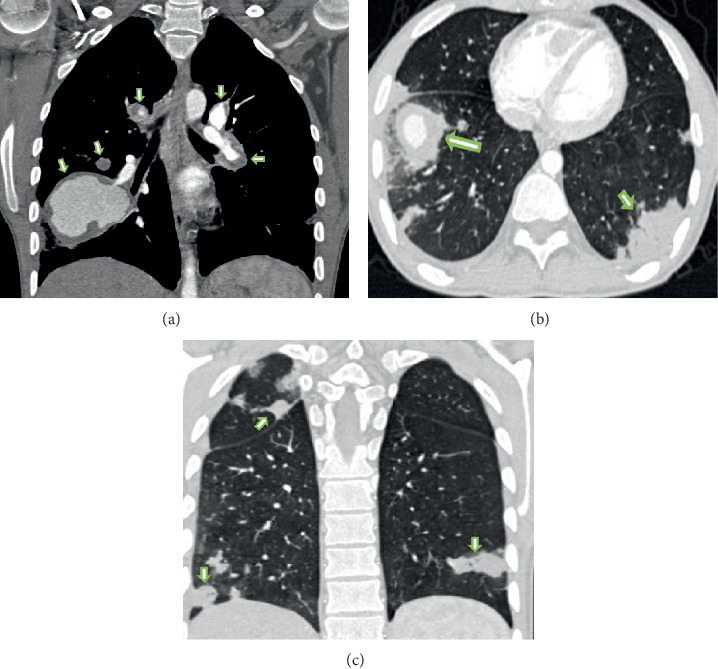
25-year-old man with multiple pulmonary artery aneurysms who had hemoptysis and chest pain with known BD. (a) Coronal maximum-intensity projection CT (curved multiplanar reconstruction) image showing multiple bilateral pulmonary artery aneurysms and mural thrombi (arrows). (b) Large right pulmonary artery aneurysm (long arrow) is partially lined by thrombus and densely opacified on a volumetric high-resolution CT. Lung window scan at the level (Figure 5(a)) shows the right pulmonary artery aneurysm with circumferential ground glass opacification because of surrounding haemorrhage (arrow): this finding was attributed to intraparenchymal haemorrhage. (c) Chest CT scans (lung window) obtained at different levels demonstrate multiple subpleural nodular infiltrations in the periphery of the right and left lung (arrows).

Pulmonary artery involvement causes pulmonary artery aneurysm (PAA), one of the most important complications of BD, and pulmonary artery thrombosis (PAT) with or without PAA [17, 48–50]. In a large series of 2500 patients performed retrospectively by Seyahi et al. [50] pulmonary artery involvement (PAI) was identified in 47 patients. %72 of the 47 patients (*n* = 34) were identified with PAA including 8 patients associated with PAT and the rest of the patients (*n* = 13, 28%) presented with isolated PAT. PAA has a particularly poor prognosis and a high mortality rate [20, 51]. Hemoptysis in varying degrees is the most common and predominant clinical manifestation [8, 20, 52]. It can be life threating when PAA is ruptured and the worst prognostic sign in BD [18, 20, 48, 51]. Moreover, PAA is associated strongly with thrombophlebitis [3, 53]. PAA or PAI associated with lung lesions such as nodules, consolidations, and cavitary lesions occurs [48, 49]. Pathologic findings suggest that arterial involvement of BD may result from neutrophilic vasculitis, which affects the vasa vasorum [54]. The arterial involvement in BD resembles those of Takayasu's arteritis, including aneurysm formation and arterial occlusion [54]. However, in BD sudden hilar enlargement or intraparenchymal round opacities on the chest radiograph or chest CT scan can present pulmonary artery aneurysm [8, 14]. PAAs may be single (Figures 2–4) or multiple unilateral or bilateral (Figure 5). These lesions may be occluded totally or partially by thrombus (Figures 2, 4, 5). Thrombosed aneurysms cause ischemia or infarction in pulmonary parenchyma [8, 20]. In BD hemoptysis can also be misdiagnosed as pulmonary thromboembolism (PTE) and this condition could be mistakenly attributed to PTE and inappropriate anticoagulation may prove fatal [3, 51]. Hemoptysis, the most frequent symptom in BD, is also caused by lung cancer, bronchiectasis, and various infectious diseases such as lung tuberculosis and fungal infections. These diseases must be included in the differential diagnosis [48]. Some studies also suggest that PAAs may show clinical and radiological regression with corticosteroid and immunosuppressive treatment [14, 52, 55]. In another study, it has been reported that aneurysms or thrombosis may disappear or regress in about 70% of the cases with only immunosuppressive treatment [50, 52]. However, the exact mechanism of this regression remains unknown [55]. Although there are reports of successful treatment results and regression of aneurysm with immunosuppressive treatment, mortality rate is still 26% [48, 49]. In BD thrombosis of the pulmonary arteries is usually in situ thrombosis secondary to vasculitis. Thrombi being organized are firmly adherent to the vascular lumens [20, 51, 56]. Although deep vein thrombosis is common in BD, PTE is uncommon [20, 57]. Hughes-Stovin syndrome (HSS) is characterized by the findings of thrombophlebitis and multiple pulmonary and/or bronchial aneurysms [58]. Behçet's disease is also associated with combination of these lesions. There is a significant overlap between the clinical, radiological, and histopathological findings of HSS and BD. Particularly pulmonary involvement is indistinguishable between two vasculitides [58]. In BD, the diagnosis is made on the basis of the criteria published in 1990 by the International Study Group for Behçet's disease [5]. There exist evident similarities between the vascular manifestations of BD and HSS. However the exact pathophysiology of both vasculitides remains unclear [58].

### 4.3. Involvement of Major Mediastinal Veins and the Superior Vena Cava

In general, vascular lesions occur in the venous system more frequently than the arterial system. The high frequency of venous involvement and the lower frequency of arterial involvement have been emphasized by different studies [45, 48, 59–62]. However, according to the literature reports in Japanese [63], North American, and European series [64], it has been reported that arterial lesions are more common than venous lesions. These studies suggest that vascular lesions show differences according to the geographical areas in BD. There is no concordance in the literature on the frequency rates of venous lesions. Major vein thromboses, such as superior and inferior vena cava, portal vein, hepatic vein, and dural sinuses, have been reported on up to 30% of the patients [45, 50, 59]. However, the most commonly venous involved site is the lower extremities followed by the SVC, the IVC, and the upper extremity veins [60, 61, 65]. In a large series of 2313 patients from Turkey, it has been reported that these venous lesions including jugular, renal, brachiocephalic, and hepatic veins occur less commonly [66].

In BD, SVC syndrome is frequently accompanied with thrombosis in SVC and rarely secondary to mediastinal fibrosis [21, 67]. Calamia KT et al. [62, 67] have reported that BD is a common cause of SVC syndrome in areas where the disease is common. In another study, Sagdıc et al. [65] have reported that BD is the most common cause of SVC syndrome in Mediterranean countries, after intrathoracic malign lesions. In BD, SVC thrombosis causes characteristic signs and symptoms of SVC syndrome [48, 50, 68]. Moreover, SVC syndrome due to vasculopathy, without evidence of thrombosis, has been presented as a case report [69]. Patients with SVC syndrome may tolerate clinical caval occlusion for many years compared to that of the malignant intrathoracic tumors. However, the deaths have been described as a result of extensive thrombosis, hemoptysis or other vascular causes, and rarely pulmonary embolus [62, 67].

**Figure 6:**
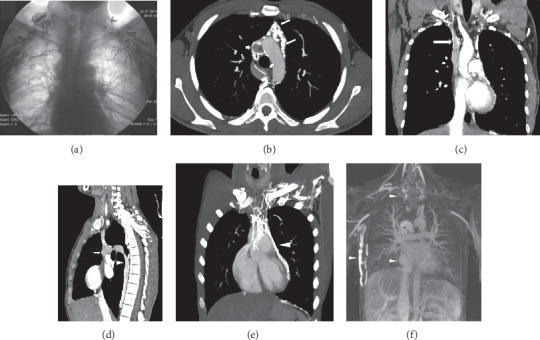
A 30-year-old male who presented with a history symptoms and signs of SVC syndrome for five years. He presented with a marked respiratory distress with cough and dyspnea. (a) Upper extremity contrast-enhanced venography shows bilateral occlusion of brachiocephalic veins and SVC. Also note the collateral veins in the chest wall and axillary region. (b) Follow-up axial contrast-enhanced chest CT scan shows nonopacification of the SVC (short arrow) and opacification of the multiple mediastinal veins (arrows). (c) Coronal maximum-intensity projection (MIP) reformation shows occlusion of the SVC due to thrombosis (long arrow) and bilaterally complete occlusion of the brachiocephalic, subclavian, and axillary veins. (d) Also, sagittal reformatted CT scan reveals that venous drainage from thorax is via the azygos system (arrow head) to SVC (arrow). (e) Coronal MIP reformation shows collateral flow through dilated pericardial vein (arrow head). (f) Coronal maximum-intensity (MIP) projection image from gadolinium-enhanced 3D MR angiography shows occlusion of the thoracic veins and SVC and dilated azygos vein (AV) and collateral veins at thoracic inlet level and chest wall (arrow heads) on the right (IVC: arrowhead). Behçet's disease was clinically diagnosed because of recurrent oral and genital ulcerations and positive pathergy test. The diagnosis was confirmed by histopathologic examination.

**Figure 7:**
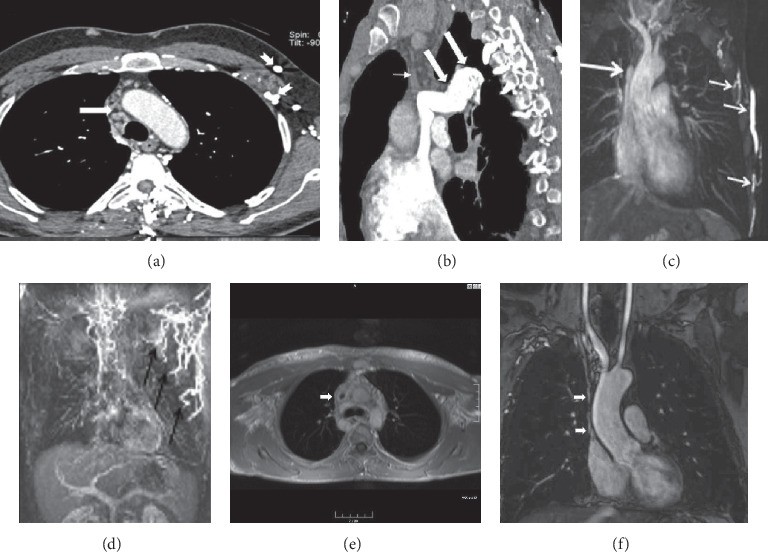
Superior vena cava syndrome in a 50-year-old man who had BD for ten years. (a) Before treatment with immunosuppressive, axial contrast-enhanced chest CT scan (mediastinal window) showing occlusion of innominate veins and SVC (thick arrow). (b) Sagittal oblique reformatted CT scan showing complete occlusion of the SVC (thin arrow) and dilated azygos vein (thick arrows). (c) Gadolinium-enhanced 3D MR venography showing bilaterally complete occlusion of major bilaterally brachiocephalic veins and SVC (long arrow) due to thrombosis and extensive collateral veins (short arrows) in the chest wall. (d) Coronal maximum-intensity images from 3D gadolinium-enhanced subtraction MR venogram obtained by subtracting from the arterial phase showing complete occlusion of brachiocephalic, subclavian, and axillar veins as well as SVC and extensive collateral circulation at chest wall on the left (black arrows). (e) After treatment with immunosuppressive, axial noncontrast 3D UTE-MRI shows SVC stenosis (arrow) and wall thickening of SVC (arrow). (f) Follow-up contrast-enhanced coronal MRA shows lack of opacification of SVC (arrows) and occlusion of bilaterally innominate veins and axillary veins after treatment with immunosuppressive.

CT diagnosis of SVC syndrome includes lack of opacification of SVC or thoracic inlet and mediastinal veins, an intraluminal filling defect in the SVC, and wall thickening and obliteration of the SVC with accompanying prominent venous collaterals in the area drained by SVC (Figures 6 and 7) [22, 32, 52]. MRI and gadolinium-enhanced MR venography also show the extent and site of mediastinal and the thoracic inlet venous occlusions and collateral veins [13, 36–38]. Chylothorax, chylopericardium, and chylous ascites are rare presenting manifestations of BD. Association of these manifestations and SVC syndrome has been presented as case reports [70, 71].

### 4.4. Involvement of the Pulmonary Parenchyma

In many patients, involvement of pulmonary parenchyma may correlate with pulmonary infections that have been reported in 0.43% of patients with BD [72]. On chest radiograph or CT scans PAAs or PAT associated with ground glass lesions (Figure 5) nodular infiltrates, consolidations, cavitary lesions, mediastinal lymphadenopathy, pleural and pericardial effusions, and intracardiac filling defects are often found [49, 50]. Subpleural nodules (Figure 5) and consolidations are commonly found during the exacerbations or at the beginning of the disease [50]. However, in a study performed in the early 2002s, Erkan et al. [20] have reported that pulmonary parenchymal findings are nonspecific. Subpleural nodular opacities or triangular opacities in CT scan are generally accepted as a foci of pulmonary hemorrhages and/or infarcts [15]. In recent studies, pulmonary parenchymal lesions such as nodular infiltrates, consolidations, ground glass opacities, and cavitary lesions have also been described in BD [48–50]. Pulmonary parenchymal lesions such as organizing pneumonia have been rarely described [26, 48, 50]. As a result, histopathologic examinations of pulmonary parenchymal lesions are necessary in the differential diagnosis of lung parenchymal lesions [49]. Pleural manifestations of BD include pleural effusions and pleural nodules. Vasculitis of pleura is often difficult to differentiate from parenchymal subpleural lesions. Pleural effusion may be attributed to pulmonary infarction, vasculitis of pleura, or SVC thrombosis [10, 14]. In BD patients with prolonged immunosuppressive treatment, bacterial, viral, fungal (Figure 4), and mycobacterial organisms may infect the lungs in a similar manner to that noted in certain types of immunosuppression [73].

### 4.5. Involvement of the Heart

**Figure 8:**
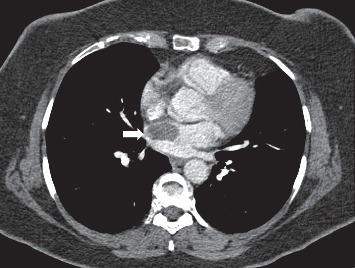
Intracardiac thrombus. A contrast-enhanced chest CT scan demonstrates a low attenuating mass-like lesion (white arrow) in the left atrium without enhancement, pathologically proven an organized clot.

Cardiac involvement occurs in 1–6% of BD patients and often is associated with pulmonary arterial and venous involvement [48, 73]. Cardiac problems in BD include pericarditis, myocarditis with conducting system findings, endocarditis with valvular regurgitation, intracardiac thrombus (ICT), coronary arteritis, and sinus of Valsalva aneurysms [48, 50, 74]. However, the authors consider that endomyocardial fibrosis in the right side of the heart is one of the causes of ICT in patients with BD without deep vein thrombus [75]. Most of the ICT in BD are seen in the right side of the heart, but left-sided and multichamber lesions have been also reported [22, 74]. The differential diagnosis of intracardiac thrombus should include primary cardiac tumors, such as haemangioma, myxoma, lymphoma, or sarcoma [29, 73]. Transesophageal echocardiography (TEE) can help in establishing the diagnosis by demonstrating a heterogeneous and echogenic mass [22]. Contrast-enhanced CT may reveal a hypodense mass or a filling defect in the ventricle as well as other thoracic manifestations such as PTE and occlusions of SVC or lung parenchymal lesions. MRI may provide diagnostic information of the ICT within the cardiac chambers and the involvement of the additional vessels or surrounding structures (Figure 8) [20, 76].

## 5. Conclusion

BD involving the chest can be characterized by aneurysms or occlusions of the aorta and its major branches or pulmonary vascular tree. Venous lesions and SVC syndrome develop after the initial diagnosis of BD in patients within long-term follow-up. CT scan is useful not only in diagnosing vascular involvement or complications but also in detecting pulmonary parenchymal involvement of BD. In comparison with catheter angiography, CT or MRA is less invasive and cost-effective and takes less time and provides diagnostic information that can help determine appropriate clinical management. Moreover, CT, MRI, MRA, and MR venography are effective imaging modalities for the diagnosis, follow-up, and evaluation of vascular complications in BD patients, without resorting to invasive procedures. Contrast-enhanced MRA (cMRA) shows inferior depiction of the lung parenchyma and may be limited in dyspneic patients due to need for breath-holding. Although gadolinium-based contrast agents are contraindicated in some patients, contrast-enhanced 3D MRA is routinely used for diagnose of thoracic arterial and venous lesions, because of its spatial resolution reliability and potential for multiplanar assessment. Larger patients studies are needed to investigate the clinical utility of free-breathing UTE-MRI. Moreover, UTE-MRI with 3 Tesla MRI system is not more readily available and expensive.

## Figures and Tables

**Table 1:** Frequency of clinical manifestations in Behçet's disease (7, 8).

Lesion	Frequency
Oral ulcers	96–100
Skin lesions	
Folliculitis 40–50	
Erythema nodosum	25–80
Positive pathergy test	10–50
Genital ulcers	65–90
Eye lesions	35–70
Arthritis	30–80
Neurological involvement	10–50
Gastrointestinal involvement	5–60
Vascular involvement	5–30
Pulmonary involvement	1–8

**Table 2:** International Study Group criteria for the diagnosis of Behçet's disease. (9) In the absence of other clinical explanations patients must have the following.

1. Recurrent oral ulceration (aphthous or herpetiform) observed by the physician or patient recurring at least three times in one 12-month period + two of the following:
2. Recurrent genital ulceration
3. Eye lesions
Anterior uveitis
Posterior uveitis (cells in the vitreous observed by slit lamp examination)
Retinal vasculitis observed by an ophthalmologist
4. Skin lesions
Erythema nodosum
Pseudofolliculitis
Papulopustular lesions or acneiform nodules in postadolescent patients not on corticosteroids
5. Positive skin pathergy test read by a physician at 48 hours—that is, a 2 mm erythematous papule or pustule at the prick site 48 hours after the application of a sterile hypodermic 20–22 gauge needle which obliquely penetrated avascular antecubital skin to a depth of 5 mm

## Abbreviations

BD: Behçet's disease
MR: Magnetic resonance
MRA: Magnetic resonance angiography
MRI: Magnetic resonance imaging
CT: Computed tomography
MSCT: Multislice computed tomography
MDCT: Multidetector computed tomography
PAA: Pulmonary artery aneurysm
3D: Three-dimensional
SVC: Superior vena cava
IVC: Inferior vena cava
PTE: Pulmonary thromboembolism
TEE: Transesophageal echocardiography
UTE: Ultrashort echo time
ICT: Intracardiac thrombus
cMRA: Contrast-enhanced MRA.
